# Ryanodine receptor 1 (*RYR1*) mutations in two patients with tubular aggregate myopathy

**DOI:** 10.1111/ejn.15728

**Published:** 2022-06-13

**Authors:** Gaetano Nicola Alfio Vattemi, Daniela Rossi, Lucia Galli, Maria Rosaria Catallo, Elia Pancheri, Giulia Marchetto, Barbara Cisterna, Manuela Malatesta, Enrico Pierantozzi, Paola Tonin, Vincenzo Sorrentino

**Affiliations:** ^1^ Department of Neurosciences, Biomedicine and Movement Sciences, Section of Clinical Neurology University of Verona Verona Italy; ^2^ Department of Molecular and Developmental Medicine, Molecular Medicine Section University of Siena Siena Italy; ^3^ Interdepartmental Program of Molecular Diagnosis and Pathogenetic Mechanisms of Rare Genetic Diseases Azienda Ospedaliero Universitaria Senese Siena Italy; ^4^ Department of Neurosciences, Biomedicine and Movement Sciences, Section of Anatomy and Histology University of Verona Verona Italy

**Keywords:** excitation–contraction coupling, ryanodine receptor, store‐operated Ca^2+^ entry, tubular aggregates

## Abstract

Two likely causative mutations in the *RYR1* gene were identified in two patients with myopathy with tubular aggregates, but no evidence of cores or core‐like pathology on muscle biopsy. These patients were clinically evaluated and underwent routine laboratory investigations, electrophysiologic tests, muscle biopsy and muscle magnetic resonance imaging (MRI). They reported stiffness of the muscles following sustained activity or cold exposure and had serum creatine kinase elevation. The identified *RYR1* mutations (p.Thr2206Met or p.Gly2434Arg, in patient 1 and patient 2, respectively) were previously identified in individuals with malignant hyperthermia susceptibility and are reported as causative according to the European Malignant Hyperthermia Group rules. To our knowledge, these data represent the first identification of causative mutations in the *RYR1* gene in patients with tubular aggregate myopathy and extend the spectrum of histological alterations caused by mutation in the *RYR1* gene.

## Graphical abstract

Identification of *RYR1* mutations uncovers the fourth gene causative of tubular aggregate myopathy.

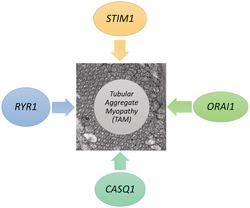

**Abbreviations**
*ASPH*
: aspartyl/asparaginyl beta‐hydroxylase encoding gene
*ATP2A1*
: ATPase sarcoplasmic/endoplasmic reticulum Ca^2+^ transporting 1 gene
*CACNA1S*
: calcium voltage‐gated channel subunit alpha1 S coding gene
*CASQ1*
: calsequestrin1 coding gene
CCD: central core disease
CFTD: congenital fibre‐type disproportion
CK: creatine kinase
CNM: centronuclear myopathy
DHPR: dihydropyridine receptor
DuCD: dusty core disease
*FXYD1*
: FXYD domain containing ion transport regulator 1 gene encoding or Phospholemman
*HRC*
: histidine rich calcium binding protein coding gene
*JPH1*
: junctophilin 1 coding gene
*JPH2*
: junctophilin 2 coding gene
*KCNA1*
: potassium voltage‐gated channel subfamily A member 1 coding gene
MHS: malignant hyperthermia susceptibility
MmD: multiminicore disease
MRI: magnetic resonance imaging
*MYH7*
: myosin heavy chain 7 coding gene
NADH‐TR: NADH‐tetrazolium reductase
*ORAI1*
: (CRAMC1) calcium release‐activated calcium channel protein 1 coding gene
*RYR1*
: ryanodine receptor 1 coding gene
SDH: succinate dehydrogenase
*SEPN1*
: selenoprotein N coding gene
SERCA: sarcoendoplasmic reticulum calcium ATPase
*SLN*
: sarcolipin coding gene
SOCE: store‐operated calcium entry
*SPEG*
: striated muscle enriched protein kinase coding gene
*SRL*
: sarcalumenin coding gene
*STAC3*
: SH3 and cysteine‐rich domain 3 gene
*STIM1*
: stromal interaction molecule 1 coding gene
TA: tubular aggregates
TAM: tubular aggregate myopathy
*TRDN*
: triadin coding gene
*TRPC3*
: transient receptor potential cation channel subfamily C member 3 coding gene

## 1 INTRODUCTION

Tubular aggregates (TA) were initially identified in human biopsies as membrane tubules, which may or not contain dense material, and have since been described in different muscle disorders (Chevessier et al., 2005; Engel et al., 1970; Schiaffino, 2012). In cryostat sections, TA appear as irregular bright red inclusions on modified Gomori trichrome technique and stain darkly with NADH‐tetrazolium reductase (NADH‐TR) but are negative for succinate dehydrogenase (SDH) staining (Chevessier et al., 2005; Schiaffino, 2012). These tubules strongly react with antibodies against sarcoplasmic reticulum proteins including SERCA1, STIM1, CASQ1 and RYR1, but also for some sarcolemmal proteins participating in Ca^2+^ signalling such as DHPR and ORAI1. At the ultrastructural level, TA appear as stacks of parallel straight tubules, arranged in a honeycomb‐like structure when observed in transverse sections (Brady et al., 2016; Schiaffino, 2012). The causal mechanisms underlying the formation of TA are not known, but they have been suggested to represent an adaptive response of the sarcoplasmic reticulum to a variety of conditions including unbalances in Ca^2+^ homeostasis, metabolic alterations, or protein aggregation and have been occasionally observed in several unrelated myopathies, which strengthen the hypothesis that they occur as a secondary non‐specific response to distressing injuries (Chevessier et al., 2004, 2005; Schiaffino, 2012).

The presence of TA in the absence of additional histopathological features identifies a distinct muscle disorder known as tubular aggregate myopathy (TAM), a rare genetic disease characterized by a wide clinical spectrum ranging from muscle weakness, myalgia and cramps to asymptomatic creatine kinase elevation. TAM often begins in childhood and may then worsen over time, although initial diagnosis in adult age is also reported. At the genetic level, TAM is predominantly caused by gain‐of‐function mutations in *STIM1* and *ORAI1* genes (Böhm et al., 2017; Böhm & Laporte, 2018; Endo et al., 2015; Lacruz & Feske, 2015; Misceo et al., 2014; Nesin et al., 2014; Silva‐Rojas et al., 2020). *STIM1* codes for a Ca^2+^ sensor localized in the sarcoplasmic reticulum, that once activated by low intraluminal Ca^2+^ levels, interacts with ORAI1 a Ca^2+^ channel localized on the plasma membrane to activate the store‐operated Ca^2+^ entry (SOCE), a mechanism that operates in all cell types to refill the intracellular Ca^2+^ stores from the extracellular environment. Given the wide expression of *ORAI1* and *STIM1*, patients with mutations in these genes may also present additional signs such as thrombocytopenia, hyposplenism, miosis and ichthyosis. The full clinical presentation of these symptoms corresponds to the Stormorken syndrome (Böhm & Laporte, 2018; Feske, 2019; Misceo et al., 2014). More recently, mutations in *CASQ1* have been also identified in some patients with TAM (Barone et al., 2017; Böhm et al., 2018). *CASQ1* codes for calsequestrin, the major Ca^2+^ binding protein in the sarcoplasmic reticulum lumen, which has also been shown to participate, together with STIM1 and ORAI1, in the regulation of SOCE (Barone et al., 2017; Shin et al., 2003; Wang et al., 2015; Zhao et al., 2010). Altogether, available data indicate that increased SOCE activity, caused by mutations in *STIM1*, *ORAI1* or *CASQ1*, represents the primary causative event of TAM. Furthermore, results from the functional characterization and a transcriptomic analysis of muscles of a mouse model carrying the pR304W *STIM1* mutation have provided evidence of the pathogenic mechanisms activated in TAM (Silva‐Rojas et al., 2021). Indeed, this study identified changes in the expression pattern of several genes encoding proteins participating in regulation of Ca^2+^ homeostasis that would contribute to the altered muscle contractile kinetics observed in these mice. In parallel, muscle fibres from *Stim1* ^R304W/+^ mice expressed higher levels ER‐stress response genes and reduced levels of some mitochondrial genes accompanied by lower oxygen consumption and ROS production. Histological analysis revealed evidence of muscle fibres degeneration and regeneration, in addition to fibres undergoing apoptosis (Silva‐Rojas et al., 2021). Overall, these results help identifying a series of events that, triggered by an increase in SOCE activity, cause structural and functional alterations that lead to the onset of myopathy.

However, in a significant number of patients with a TAM diagnosis, mutations in *STIM1*, *ORAI1* or *CASQ1* are not present, leaving these cases without a molecular genetic diagnosis.

The ryanodine receptor type 1 (*RYR1*) gene codes for the Ca^2+^ release channel of the sarcoplasmic reticulum in skeletal muscle cells (Meissner, 2017). Mutations in *RYR1* were initially identified in individuals with Malignant Hyperthermia Susceptibility (MHS), a pharmacogenetic disorder triggered by volatile anaesthetics and succinylcholine (Lawal et al., 2020). Shortly thereafter, *RYR1* mutations were identified in patients with central core disease (CCD) and in other myopathies collectively referred to as *RYR1*‐related myopathies which include multiminicore disease (MmD), centronuclear myopathy (CNM), congenital fibre‐type disproportion (CFTD) and dusty core disease (DuCD). These diseases are classified based on the presence, at the histological analysis, *of distinctive structures like* central cores, minicore, nemaline rods, fibre‐type disproportion and dusty cores (Lawal et al., 2018). *RYR1*‐related myopathies are generally non‐progressive or slowly progressive and are characterized by a wide range of symptoms including mild muscle weakness, hypotonia, motor developmental delay, orthopaedic complications, including scoliosis and foot deformities, and, more rarely, to cases with wheelchair dependence and respiratory failure (Dowling et al., 2014; Jungbluth et al., 2018; Lawal et al., 2020). More recently, *RYR1* variants have also been associated with other atypical phenotypes including exercise‐induced rhabdomyolysis (Knuiman et al., 2019), some forms of periodic paralysis (Jungbluth & Hanna, 2018), adult‐onset distal myopathy (Machnicki et al., 2021; Pietrini et al., 2004; Zhou et al., 2010), mild calf‐predominant myopathy (Jokela et al., 2019), foetal akinesia deformation sequence syndrome/arthrogryposis multiplex congenital and lethal multiple pterygium syndrome (Alkhunaizi et al., 2019 **)**.

Here, we report on the identification of causative dominant *RYR1* variants in two patients with a history of myopathy characterized by the presence of tubular aggregates in muscle biopsy and negative for mutations in *STIM11, ORAI1* and *CASQ1*.

## 2 MATERIALS AND METHODS

### 2.1 Muscle biopsy staining

Muscle biopsies from *vastus lateralis* muscle were performed for diagnostic purposes after written informed consent. Muscle samples were snap frozen in liquid nitrogen‐cooled isopentane and stored at −80°C. Serial 10‐μm‐thick cryosections were stained with haematoxylin and eosin, modified Gomori trichrome, adenosine triphosphatase (ATPase, pre‐incubation at pH 4.3, 4.6 and 9.4), nicotinamide adenine dinucleotide tetrazolium reductase (NADH‐TR) and succinate dehydrogenase (SDH).

A small fragment of muscle tissue from patient 1 was fixed in 4% glutaraldehyde in phosphate buffer, post‐fixed in 2% osmium tetroxide, dehydrated and embedded in Spurr resin. Ultrathin sections were stained with uranyl acetate and lead citrate and examined with a Philips Morgagni transmission electron microscope (FEI Company Italia Srl, Milan, Italy) operating at 80 kV and equipped with a Megaview II camera for digital image acquisition.

Biochemical analysis of glycolytic enzymes including myophosphorylase, phosphofructokinase, phosphoglycerate kinase, phosphoglycerate mutase and lactate dehydrogenase was performed on muscle homogenates, as previously described (Filosto et al., 2007).

Immunohistochemistry was done on serial 8‐μm‐thick sections with antibodies to sarcoplasmic/endoplasmic reticulum Ca^2+^ ATPase 1 (SERCA1) (1:500; Santa Cruz Biotechnology), sarcoplasmic/endoplasmic reticulum Ca^2+^ ATPase 2 (SERCA2) (1:100, Santa Cruz Biotechnology), stromal interaction molecule 1 (STIM1) (1:100, Abcam) and ryanodine receptor 1 (RYR1) (1:500; Rossi et al., 2014). Immunofluorescence was performed as previously described (Guglielmi et al., 2013). Image acquisition was performed with an Axiolab fluorescence microscope equipped with an AxioCam HRm digital camera (Carl Zeiss).

### 2.2 Genetics

Mutation screening was performed by Next Generation Sequencing technology using the *Ion GeneStudio S5 System* technology (Thermo Fisher Scientific) and the Ion Ampliseq Designer software (Thermo Fisher Scientific) to design a multiexon amplicon panel containing a total of 20 genes known to be associated with myopathies and including *RYR1*, *CACNA1S*, *MYH7*, *SEPN1*, *ATP2A1*, *STAC3*, *ASPH*, *TRDN*, *KCNA1*, *TRPC3*, *HRC*, *JPH1*, *JPH2*, *CASQ1*, *STIM1*, *ORAI1*, *FXYD1*, *SLN*, *SPEG* and *SRL*. Gene coverage of this panel was >99%. To analyse the data obtained, a routine bioinformatic pipeline that adopts the S5 Torrent Server VM was applied (Thermo Fisher Scientific). Identified variants were validated by PCR‐based standard capillary Sanger sequencing. Mutations were annotated based on *RYR1* transcript (NM_000540.3 GRCh37).

## 3 RESULTS

### 3.1 Patient description

#### 3.1.1 Patient 1

A 30‐year‐old Caucasian (Italian) man was referred for asthenia and persistent increase of serum creatine kinase (CK) levels (2‐ to 4‐folds the normal values) started 8 years before. Since childhood he complained of hand stiffness with repetitive movements and during exposure to cold. He denied cardiac or respiratory problems. His father had mild serum CK increase (2‐fold above the control value) and from 10 years complained of muscle cramps. On clinical evaluation, the patient had a normal gait and could easily get up from a chair or squat. Muscle strength and sensation were normal at four limbs as well as deep tendon reflexes. Cranial nerves examination showed a mild right eyelid ptosis. Routine laboratory tests and muscle MRI were unremarkable, while needle electromyography documented myopathic changes at four limbs. At age 35, the patient referred the same symptoms, and the neurological examination was unchanged.

#### 3.1.2 Patient 2

A 39‐year‐old Caucasian (Italian) man came to our attention because of persistent CK increase (2–4 times normal values). From early adulthood, he complained of muscle stiffness that get**s** worse with repeated movements and after cold exposure. At age 20 he experienced a single episode of pigmenturia after strenuous physical effort, however biochemical analysis of glycolytic enzymes ruled out a metabolic myopathy. Neurological examination was normal, and no muscle weakness was observed. Routine laboratory tests, nerve conduction studies and needle electromyography were normal. The patient's symptoms and neurological examination did not change during the 8‐year follow‐up.

### 3.2 Pathological findings

**FIGURE 1:**
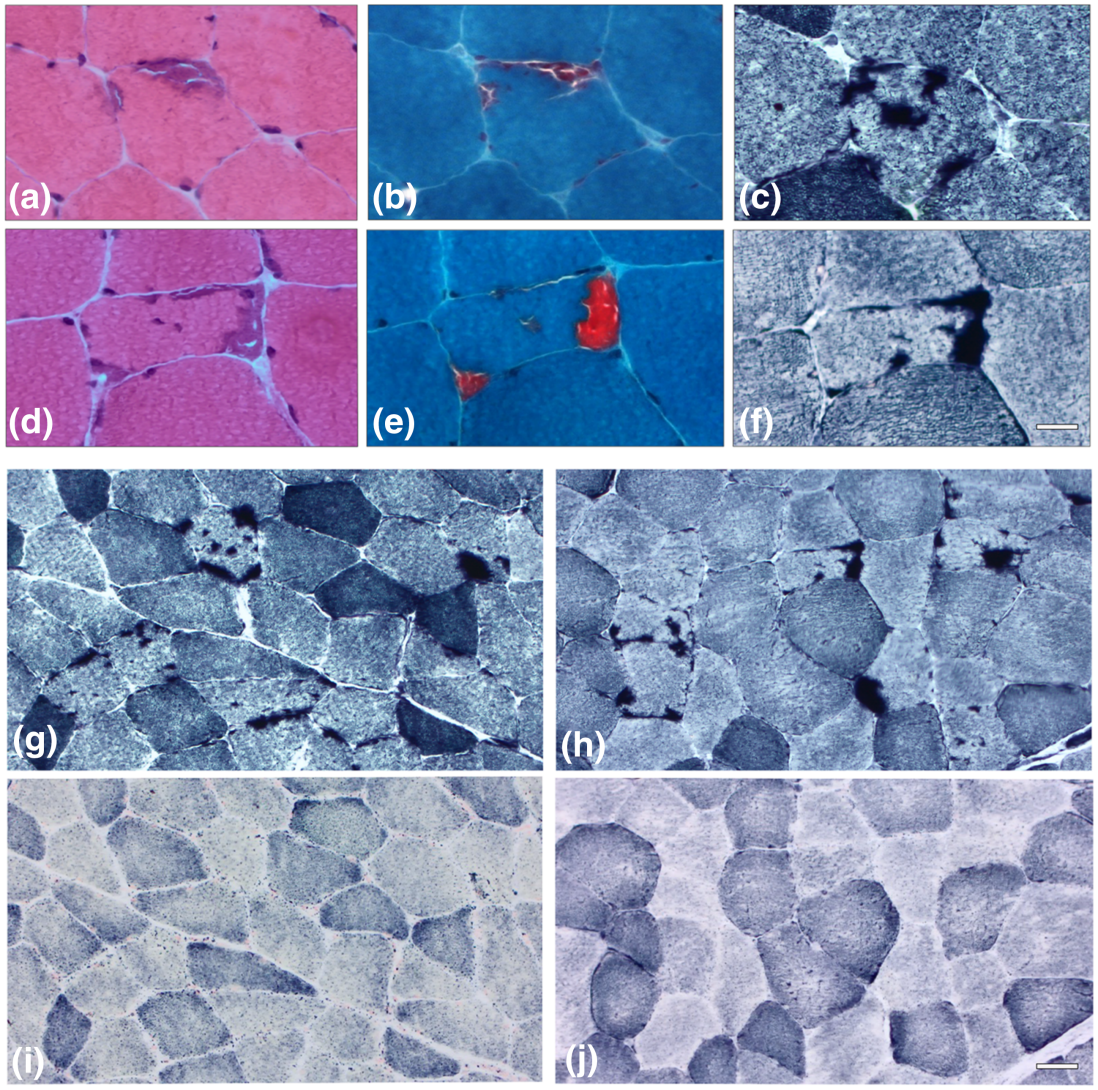
Histochemical staining of muscle biopsies. Muscle biopsies from patient 1 (a–c) and patient 2 (d–f) show a muscle fibre with tubular aggregates which appear basophilic on haematoxylin and eosin (a, d), stain bright red with the modified Gomori trichrome (b, e) and dark blue with NADH‐TR (c, f). Muscle biopsies from patient 1 (g, i) and patient 2 (h, j) show several muscle fibres with multiple tubular aggregates strongly reactive in NADH‐TR staining (g and h) and negative to SDH reaction (i and j). Bar: 20 μm (a–f); bar: 50 μm (g–j)

**FIGURE 2:**
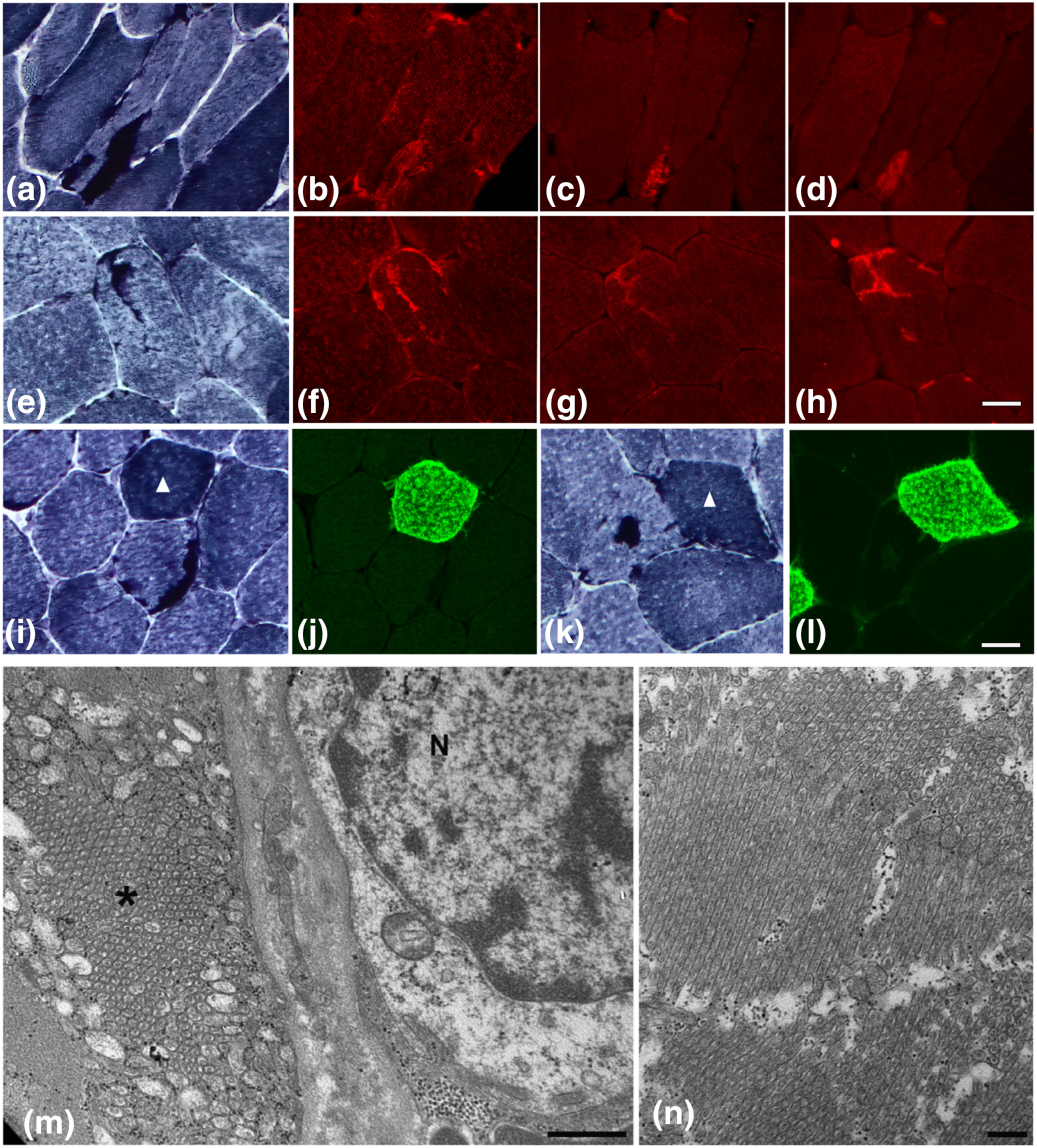
Characterization of tubular aggregates. NADH‐TR and immunofluorescence staining for SERCA1, RYR1 and STIM1 (a–h). Muscle biopsy from patient 1 (a–d) and patient 2 (e–h) were stained with NADH‐TR (a, e) and decorated with antibodies for SERCA1 (b–f), RYR1 (c, g) and STIM1 (d, h). Images were obtained with objective ×40. Bar: 20 μm. NADH‐TR and immunofluorescence staining for SERCA2. Muscle biopsies from patient 1 (i–j) and patient 2 (k–l). No signal for SERCA2 (j, l) was detected in tubular aggregates found in type 2 muscle fibres (I, K; NADH‐TR). Arrowheads point to type 1 muscle fibres. Images were obtained with objective ×20, scale bar: 20 μm. Transmission electron micrographs of muscle biopsy of patient 1 (m–n). Electron microscopy analysis of muscle fibres from patient 1 shows large tubular aggregates (m, n). In (n), the aggregate is composed of bundles of tubules running in various directions. N, nucleus; *, tubular aggregate. Bars: 500 nm (m), 200 nm (n)

In both patients, muscle biopsy showed the presence of granular inclusions with the distinctive histochemical features of tubular aggregates in 4 and 10% of muscle fibres from patient 1 and patient 2, respectively (Figure 1). Tubular aggregates were often multiple and almost exclusively located in the subsarcolemmal area of type 2 muscle fibres. Increased fibre size variation and reduced number of type 1 fibres were also observed. Immunostaining of serial cross‐sections from patients' biopsies with antibodies against SERCA1 (Figure 2b,f), STIM1 (Figure 2c,g) and RYR1 (Figure 2d,h) confirmed the SR origin of these aggregates. In agreement with preferential presence in type 2 fibres, no signal was observed with antibody to SERCA2 in muscle fibres presenting tubular aggregates (Figure 2i–l). Electron microscopy analysis was performed on specimen from patient 1 and confirmed the presence of single or multiple areas with large bundles of parallel membrane tubules (Figure 2m,n). No muscle tissue was available from patient 2 for ultrastructural investigations. Activity of glycolytic enzymes was normal in both patients (data not shown).

### 3.3 Genetic analysis findings

Sanger sequencing excluded the presence of mutations in *STIM1*, *ORAI1* and *CASQ1* coding sequences in the two patients. Genetic analysis was performed using a targeted next generation sequencing (NGS) panel including 20 genes participating in regulation of excitation‐contraction coupling/Ca^2+^ signalling in skeletal muscle and known to be causative of myopathy. This analysis resulted in the identification of causative variants in the *RYR1* gene in these two patients. In patient 1, we identified a previously reported missense variant in *RYR1*, c.6617C > T in exon 40 (MAF 1:47102). This variant results in the substitution of a threonine with a methionine at codon 2206 (p.Thr2206Met). The p.Thr2206Met mutation has been previously associated with MHS (Carpenter et al., 2009; Wehner et al., 2002) and is included in the list of causative mutations by the EMHG (https://www.emhg.org). This mutation is located in the bridging solenoid, a domain of the RYR1 channel structure where most causative mutations are located (des Georges et al., 2016; Woll et al., 2021; Woll & Van Petegem, 2022). In patient 1, a second variant, c.2371C > A in exon 20 (MAF 1:83,830), was identified in the *RYR1* gene. This variant results in the substitution of a leucine with an isoleucine at codon 791 (p.Leu791Ile). The L791 residue is localized within the core of the Sp1A kinase‐ryanodine receptor (SPRY1) domain of RYR1 (des Georges et al., 2016). The function of this domain has not been completely defined. In silico analysis of the p.Leu791Ile variant was ambiguous, since, according to PolyPhen‐2 it is “probably damaging” (score 0.999), but with other programs the score is less severe (SIFT: 0.088; Provean: −1.79; REVEL: 0.468). Accordingly, this second variant is to be considered benign.

In patient 2, we also identified a known causative heterozygous variant in *RYR1*. This variant, c.7300G > A in exon 45 (MAF 1:25,696) results in the amino acid substitution of a glycine with an arginine at codon 2434 (p.Gly2434Arg) (Murayama et al., 2016). This variant is considered pathogenic and causative for MHS based on the EMHG guidelines. The pGly2434Arg mutation is also located in the bridging solenoid. Patient 2 also carries a second heterozygous missense variant, c.10747G > C in exon 73 of *RYR1*. This variant results in the amino acid substitution of a glutamic acid for a glutamine at codon 3583 (p.Glu3583Gln) a residue located in the bridging solenoid domain. However, considering the high frequency (MAF 1:68) and the results of software for in silico prediction of pathogenicity (PolyPhen: 0.532; SIFT: 0.23; Provean: −0.75; REVEL: 0.32), this variant is considered benign.

## 4 DISCUSSION

Here we report the first identification of mutations in *RYR1* in two unrelated patients with clinical symptoms of a mild myopathy and the presence of tubular aggregates as the sole pathological abnormality on muscle biopsy. In the absence of symptoms reminiscent of a specific form of myopathy and because of morphological features, they were diagnosed as having TAM. Both patients had a mild increase in serum CK levels and reported episodic stiffness triggered by repetitive muscle contraction or exposure to cold, a clinical phenotype not typical for TAM. Patient 2 also experienced a single episode of pigmenturia in his youth after strenuous physical effort; biochemical analysis of glycolytic enzymes was performed to rule out a metabolic myopathy. Indeed, type X glycogenosis was reported in patients whose muscle biopsy showed tubular aggregates (Naini et al., 2009; Vissing et al., 1999). Identification of mutations in *RYR1* represents an unexpected finding, since myopathies caused by *RYR1* mutations are usually associated with the presence of cores of different morphologies in muscle biopsy of these patients (Knuiman et al., 2019; Lawal et al., 2020). Indeed, despite in the last years the list of *RYR1*‐related myopathies has been further expanded with the inclusion of novel histological findings such as dusty cores (Garibaldi et al., 2019) and protein aggregate inclusions (Machnicki et al., 2021), *RYR1* mutations have never been identified in patients with TAM. In the past, patients with exercise‐related transient muscle stiffness and TA (Müller et al., 2001) and a single case report of MHS with tubular aggregates in muscle biopsy have been reported (Reske‐Nielsen et al., 1975), but no genetic diagnosis was available for those patients. Even though both patients carry a second variant (p.Leu791Ile or p.Glu3583Gln, in patient 1 and patient 2, respectively), these additional variants are considered benign based on literature reports, frequency in gnomAD data base and the results from software for in silico prediction of pathogenicity. Accordingly, considering the results of the genetic analysis and that the father of patient 1 reported similar symptoms, the disease observed in these two patients appears to have a dominant inheritance pattern.

Of note, both patients are carriers of causative mutations (p.Thr2206Met or p.Gly2434Arg, in patient 1 and patient 2, respectively) that previously were mainly detected in MHS individuals, which makes them susceptible to MH crisis, although they have not report the occurrence of MH episodes in their families. The p.Thr2206Met mutation, identified in patient 1, was predominantly associated with the MHS phenotype and shown, by in vitro functional characterization, to exhibit an enhanced sensitivity to caffeine and 4‐chloro‐cresol, a reduced sarcoplasmic reticulum Ca^2+^ content, and a small increase in resting cytoplasmic Ca^2+^ level (Murayama et al., 2016; Wehner et al., 2002). The p.Thr2206Met mutation was also reported, in a homozygous state, in a patient with CCD (Garibaldi et al., 2019) and in a family with CCD where it was in association with an additional variant potentially affecting the splicing processing of *RYR1* mRNA (Snoeck et al., 2015). The p.Gly2434Arg identified in patient 2 represents the causative mutation most frequently associated with MHS in the UK (Robinson et al., 2006) and with exertional heat stroke (Butala & Brandom, 2017; Kraeva et al., 2017). Accordingly, mice carrying the Gly2434Arg mutation showed an increase in death rate following exposure to halothane or to increased ambient temperature, indicating that this mutation is potentially associated to environmental heat stroke (Lopez et al., 2018). Functional characterization of the p.Gly2434Arg mutation showed an increased sensitivity to caffeine and 4‐chloro‐m‐cresol (Richter et al., 1997). Both p.Thr2206Met and p.Gly2434Arg mutations are located in the bridging solenoid, a domain of RYR1 structure where several causative mutations are found (des Georges et al., 2016; Woll et al., 2021; Woll & Van Petegem, 2022).

Certainly, the finding that mutations mostly associated to MHS may also cause a myopathy with TA, represents an intriguing question. On the other hand, it is known that RYR1 mutations, although present in different regions of the RYR1 sequence (Amburgey et al., 2013; Galli et al., 2006; Robinson et al., 2006) do not show any correlation between mutation location, RyR1 channel activity and clinical phenotype (Amburgey et al., 2013; Todd et al., 2018). An additional intriguing aspect emerging from the data we are reporting is that the allele frequency in the general population of the two causative *RYR1* mutations (1:47,102 and 1:25,696 for p.Thr2206Met and p.Gly2434Arg, respectively) is much higher than the frequency of a very rare disease such as TAM (Conte et al., 2021; Silva‐Rojas et al., 2020). This apparent contradiction could be, at least in part, explained by considering that causative RYR1 mutations may have reduced penetrance (Ibarra Moreno et al., 2019; Shaw & Hopkins, 2019). However, it is important to note that the two TAM patients reported here have a mild phenotype compared to the more severe phenotype of most TAM patients carrying mutations in *STIM1* and *ORAI1*. The latter observation therefore suggests the possibility that the frequency of RYR1‐related TAM can be underestimated. Therefore, even considering that we sequenced only 20 genes, all of the above suggest that the Gly2434Arg and p.Thr2206Met mutations are likely causative of the observed tubular aggregates myopathy observed in these two patients.

## 5 CONCLUSION

In conclusion, these findings reinforce the view that, regardless that the mutated genes encode proteins that participate in SOCE or excitation‐contraction coupling mechanisms, altered Ca^2+^ homeostasis represents a key event in the development of myopathies, even though there is a wide variability in clinical symptoms and histological features induced by mutations in these genes.

The identification of mutations in *RYR1* in two patients with TAM further extends the number of TAM‐related genes and provides additional evidence that mutations in different genes may converge in inducing the development of tubular aggregates. Accordingly, these data suggest that *RYR1* should be considered for genetic analysis in a myopathy with TA where *STIM1*, *ORAI1* and *CASQ1* mutations have been excluded. On the other hand, these results also expand the spectrum of non‐core myopathies within the larger family of *RYR1*‐related myopathies.

## CONFLICT OF INTEREST

The authors declare that they have no conflict of interest.

## AUTHOR CONTRIBUTIONS

GNAV, DR and VS conceived the article and wrote the manuscript. DR, LG, MRC, EP, GM, BC, MM, EP and PT collected the data and performed experiments. GNAV, DR and VS reviewed and edited the manuscript. All authors reviewed the manuscript.

### PEER REVIEW

The peer review history for this article is available at https://publons.com/publon/10.1111/ejn.15728.

## Data Availability

The data supporting the findings of this study are available from the corresponding author upon reasonable request.
